# Long-Term Outcomes and Survival in Patients Undergoing Multiple vs. Single Renal Artery Transplants: A Retrospective Cohort Study

**DOI:** 10.7759/cureus.78165

**Published:** 2025-01-28

**Authors:** Karthik Kanna Venkatesh, Sreerag Sreenivasan Kodakkattil, Sreejith Parameswaran, KM Abdulbasith, Sidhartha Kalra, Dorairajan Lalgudi Narayanan, Deepanshu Aggarwal, Jithesh P

**Affiliations:** 1 Medicine, Jawaharlal Institute of Postgraduate Medical Education & Research, Puducherry, IND; 2 Urology and Renal Transplantation, Jawaharlal Institute of Postgraduate Medical Education & Research, Puducherry, IND; 3 Nephrology, Jawaharlal Institute of Postgraduate Medical Education & Research, Puducherry, IND; 4 Community Medicine, Jawaharlal Institute of Postgraduate Medical Education & Research, Puducherry, IND

**Keywords:** kidney transplant, multiple renal artery, post-transplant complications, post-transplant outcomes, renal transplant, single renal artery

## Abstract

Introduction

The incidence of end-stage renal disease (ESRD) is increasing, and strategies are needed to expand the available kidney donor pool. Urologists have not preferred multiple renal artery (MRA) grafts due to concerns about higher complication rates. This study aimed to compare graft and patient survival post-transplant, complications, and the time taken to reach the nadir creatinine levels.

Methods

The records of 185 patients who had undergone renal transplants in a tertiary care center in Puducherry from 2012 to 2021 were collected and were divided into two groups: Single renal artery (SRA) graft recipients in group 1 and MRA graft recipients in group 2, and was further subdivided into deceased and live donor grafts, and further data were retrieved from the case sheets, master chart maintained by the Nephrology and Urology departments and Healthcare Information System (HIS) web portal.

Results

The mean age of group 1 was 33.54±10.44 and for group 2 it was 33.18±10.32 years, gender distribution and BMI of the two groups were also similar. The two-year graft survival for the SRA group was 76.06%, and for the MRA group was 81.25%. For the two years of patient survival, the SRA group had 85.43% survival and the MRA group had 81.25% survival. The creatinine returned to nadir level by day 5 to day 7 post-transplant for both groups. Also, the development of various complications in the post-transplant period was similar in both groups; however, the MRA group who underwent deceased donor transplants faced a higher risk of complications of the transplant operation.

Conclusion

This study demonstrates that kidney transplants using MRA grafts yield comparable outcomes to SRA grafts, despite challenges such as longer ischemia times and higher complication rates. Larger, multi-center studies are needed to further evaluate MRA grafts and optimize surgical approaches for broader donor inclusion.

## Introduction

End-stage renal disease (ESRD) is the advanced stage of chronic kidney disease (CKD), where kidney function is so low that the patient cannot survive in the absence of renal replacement therapy [1]. The prevalence of CKD in India is high, with hypertension and diabetes being the most common risk factors in the Indian setting [2,3].

ESRD patients are usually dependent on dialysis or renal transplantation for long-term survival. At times, the transplant procedure becomes complicated due to anatomic variants of renal arteries, with multiple renal arteries (MRAs) being the most common one [4]. MRA occurs unilaterally in 23% and bilaterally in 10% of people, and there is an increasing need to include such kidneys to expand the potential donor pool [5].

Grafts with MRAs in deceased donors (DDs) can be managed with an aortic cuff with arteries, which is not possible in living donations. In addition, grafts with MRA tend to have delayed graft function (DGF), complications involved in vascular anastomosis, prolonged ischemia time, and poorly controlled hypertension from segmental infarctions of the allograft. Despite these drawbacks, studies have proposed that renal transplants of allografts with MRAs are on par with SRA donor kidneys in the safety aspect [5,6].

During the literature search, we found a paucity of studies related to this aspect of renal transplantation from the Indian subcontinent, especially from the southern side. The existing literature does not explain much about the influence of MRA on transplant outcomes, and contradictory results have been reported in the literature [7]. This study specifically analyzes the situation in an emerging tertiary care center in Puducherry, India. The primary objective of this study was to compare the time taken post-transplant, for the return of serum creatinine (Scr) value to nadir level (Scr<1.0mg/dL). The secondary objectives of this study were to evaluate graft survival, patient survival, and short- and long-term complications that developed in each group and subgroup.

## Materials and methods

A retrospective cohort study design was adopted for this study. All renal transplants performed between March 2012 and April 2021 were recruited for the study, with the exclusion criterion being the unavailability of necessary data. The hospital’s renal transplantation program had started in 2012, and 233 transplants had been done until April 2021. The recipients were divided into two groups based on the multiplicity of renal arteries present in the harvested graft: Group 1, graft with a single renal artery (SRA), and Group 2, graft with MRA. Patients in each of the two groups were subgrouped based on the status of the donor at the time of organ harvest: live donor (LD) and DD.

Procedure for renal transplantation in the study setting: grafts were obtained from both deceased and living donors. All living donors underwent open or laparoscopic nephrectomy. Open surgeries were preferred for right-sided kidney grafts initially, but at present, irrespective of the side laparoscopic retrieval is being done. Hem-o-lok clips (Advin Health Care, Ahmedabad, India) were used to ligate the renal artery and vein. SRA anastomosis was performed on the external iliac artery (EIA) using a parachuting technique. The technique for MRA anastomosis is performed depending on the positioning and size of the renal arteries. If almost equal in size and could be brought nearby, a pantaloon technique of joining both arteries was performed and anastomosed to the EIA. An end-to-side technique was used when the accessory artery was smaller and near the main artery. If the arteries were far apart, both were anastomosed separately to EIA.

After obtaining approval from the institute ethics committee (project no: JIP/IEC-OS/2023/132), data retrieval was performed from June 2023 to August 2023. Written consent was obtained from the patients before the transplant procedure; otherwise, no consent was required for this study because a waiver of consent was issued. All authors adhered to the ethical guidelines of the Declaration of Helsinki and its amendments throughout this study. The authors of this study have access to the original data reported in this study. Records of kidney transplant recipients, including pre-transplant evaluation data of recipients and donors, discharge summaries, and follow-up visit data, were retrieved from the database maintained by the transplant coordinators. Laboratory data were retrieved from the hospital information system. Data were collected and entered into a pre-designed data collection form and later entered into an MS Excel file (Microsoft Corporation, Washington, DC). The primary outcomes for this study were Scr values (on postoperative day-0/5/7 and at the time of discharge), cold and warm ischemia time (WIT), and patient and graft survival (short-term and long-term), other outcomes of the study are mentioned in Table 1. The time from clamping of the aorta or renal artery to cold perfusion was considered the WIT. In contrast, the cold ischemia time (CIT) was the time from cold perfusion of the kidney to the release of the renal artery and renal vein clamps [8]. DGF is defined as grafts in which Scr does not reach the nadir level in one week and requires at least one hemodialysis session in the first week post-transplantation. Slow graft function (SGF) was used for grafts that did not attain the nadir creatinine level (1.0mg/dL) by one week post-transplant but did not require dialysis.

**Table 1:** List of variables studied Scr - Serum Creatinine, SRA - Single Renal Artery Grafts, MRA - Multiple Renal Artery Grafts, UTI - Urinary Tract Infection

Independent variables	Dependent variables
1. Age (years)	1. Warm ischemia time (in minutes)
2. Marital status (single/ married/ widowed or separated)	2. Cold ischemia time (in minutes)
3. Comorbidities (diabetes/hypertension/heart disease/liver disease/others)	3. Scr values (mg/dL) (on postoperative day-0/5/7 and at the time of discharge)
4. Body mass index (underweight/normal/overweight/obese)	4. Graft survival (end of 3^rd^-month/6^th^-month/1-year/2-years) (yes/no)
5. Anatomical variation in arterial supply of donor kidney (SRA/MRA)	5. Patient survival (end of 3^rd^-month/6^th^-month/1-year/2-years) (yes/no)
	6. Post-renal transplant infections (UTI/cytomegalovirus/BK virus/others)
	7. Urological complications (hydronephrosis/urine leak/others)
	8. Vascular complications (vessel thrombus/perirenal hematoma/lymphocele)
	9. Repeat surgery due to complication (yes/no)
	10. Delayed graft function (yes/no)
	11. Incisional hernia (yes/no)

The sample size was calculated on the basis of the mean creatinine values in both the groups post-transplant using the formula for comparison of means. As only 32 MRA transplant data were available, the final sample included 185 (SRA=153; MRA=32) [9]. Further, we attempted sub-group analysis, and the final sample included: SRA with LD=90; SRA with DD=63; MRA with LD=24; and MRA with DD=8.

Analysis was performed using STATA v14 (StataCorp, College Station, TX). Comparison of continuous variables between the SRA and MRA groups was performed using the independent t-test or Mann-Whitney U test based on the distribution of variables. We compared the characteristics of subgroups (deceased and living donors) of SRA and MRA transplants using ANOVA or Kruskal-Wallis tests depending on the distribution. The statistical significance of categorical variables was tested using the chi-square test or Fischer’s exact test. Further Kaplan-Meier analysis was done to plot survival curves. Multi-level mixed effects regression was attempted to quantify the effect estimate for factors related to patient survival, graft survival, and serum creatinine values (Table 2). P-value < 0.05 was considered statistically significant.

**Table 2:** Multi-level mixed-effects regression analysis of serum creatinine, patient survival, and graft survival OR - Odds Ratio, Ref - Reference, SRA - Single Renal Artery Group, MRA - Multiple Renal Artery Group * Odds ratio was found using multi-level mixed effects logistic regression, † Adjusted for age and gender, ‡  β-coefficient estimated through multi-level mixed effect linear regression

Type of graft	Graft survival		Patient survival		Serum creatinine	
	OR*	Adjusted OR*^†^	OR*	Adjusted OR*^†^	β-coefficient	Adjusted β-coefficient^‡†^
SRA	Ref	Ref	Ref	Ref	Ref	Ref
MRA	1.185 (1.03-1.35)	1.16 (1-1.34)	0.85 (0.58-1.26)	0.84 (0.56-1.25)	-8.92 (-37.93-20.08)	-8.44 (-37.45-20.56)

## Results

A total of 185 kidney transplantation operations that were performed during the study period were analyzed for this study. The baseline characteristics of the patients are provided in Table 3. On comparing the mean age of the two groups, 33.5±10.4 years in SRA and 33.2±10.3 years in MRA (p=0.85), there was not much variation found. The mean BMI of group 1 was 20.4±4.0 m/kg^2^ and in MRA 19.9±3.5 (p=0.44).

**Table 3:** Characteristics of patients who have undergone renal transplants (single artery and multiple artery) in the last 10 years in a tertiary care center in Puducherry, India N= 185 *Independent t-test, †Chi-Squared, ‡Fisher exact

Patient characteristics	Overall (N=185)	Single renal artery graft (n=153)		Multiple renal artery grafts (n=32)		P-value
Age(years)	33.48±10.39	33.54±10.44		33.18±10.32		0.85*
BMI (kg/m^2^)	20.32±3.88	20.41±3.96		19.87±3.48		0.44*
Gender	-	Male	Female	Male	Female	0.724^†^
		77.78%	22.22%	80.65%	19.35%	
Comorbidities						
Hypertension	148	122 (79.74%)		26 (81.25%)		0.846^†^
Diabetes mellitus	17	12 (7.84%)		5 (15.63%)		0.166^†^
Hyperparathyroidism	11	10 (6.54%)		1 (3.13%)		0.693^‡^

The commonly observed preexisting comorbidities in patients with ESRD were as follows: hypertension (80%), diabetes mellitus (8.1%), and hyperparathyroidism (5.9%). Some common etiologies for ESRD observed in our study were IgA nephropathy (16 patients), chronic glomerulonephritis (CGN), focal segmental glomerulosclerosis (FSGS), and malignant nephrosclerosis. Table 3 shows the prevalence of comorbidities in both groups.

Five patients underwent pantaloon reconstruction while two patients underwent end-to-side reconstruction, which was anastomosed to EIA. A patient with four arteries underwent a pantaloon technique for the hilar vessels, which was anastomosed to the internal iliac artery (IIA) and separate anastomosis for both the polar arteries to EIA. A triple vessel graft obtained from DD underwent anastomosis of the hilar artery to the IIA and two polar vessels with the EIA.

**Figure 1:**
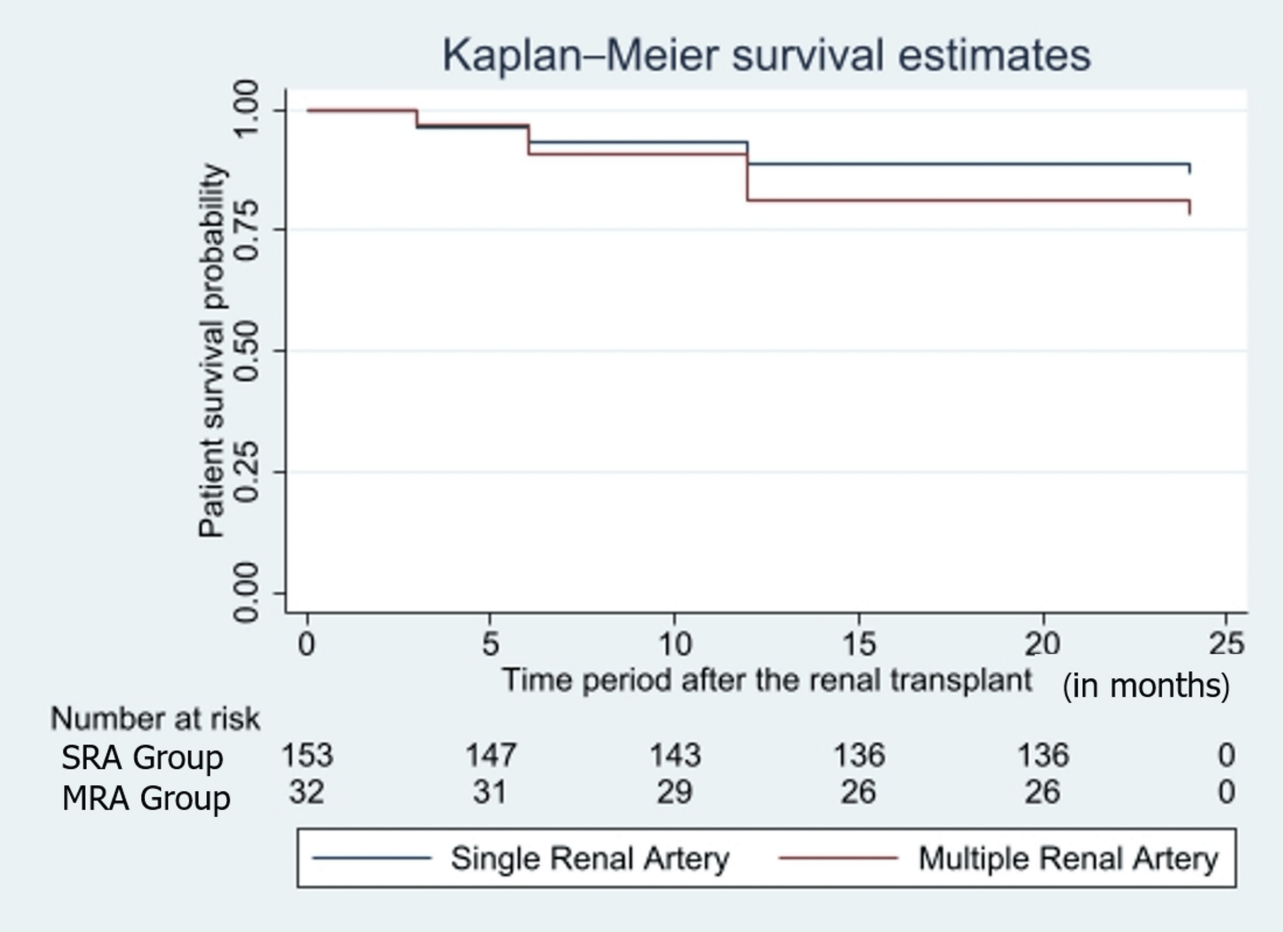
Kaplan-Meier graph representing the patient survival in both groups

**Figure 2:**
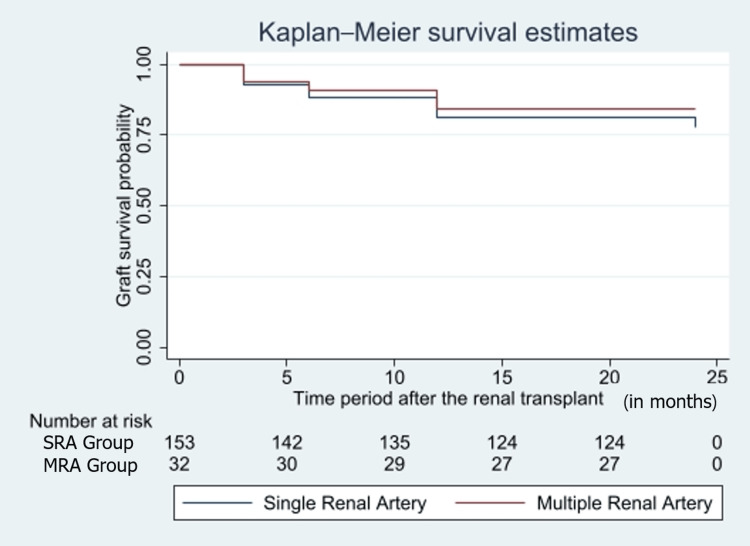
Kaplan-Meier graph representing the survival rates of the SRA and MRA groups

Statistically significant differences were found when perioperative characteristics were compared, the MRA group had longer warm and CIT (p=0.0001) (Table 4). The MRA group showed an increase in median Scr from 1.35mg/dL on postoperative day 5 to 1.45mg/dL on postoperative day 7. The long-term outcomes were compared based on the post-operative graft and patient survival rates. The patient survival rate was found to be better in the SRA group (85.43% (SRA) vs 81.25% (MRA)) (p=0.172). A contrasting trend was seen in graft survival rate with the MRA grafts (81.25%) having a higher survival rate than SRA (76.06%) (adjusted OR=1.16 (adjusted for age and gender)) (p=0.159), but the infectious and vascular complication arising due to the surgery were seen at higher rates in MRA (Figures 1, 2).

**Table 4:** Comparison of intra and postoperative characteristics in both groups SRA - Single Renal Artery group, MRA - Multiple Renal Artery group * Kruskal Wallis, †Fisher’s Exact, ‡ Chi-Squared test

Intra-operative parameters	Overall	SRA			MRA			P-value*
		Live donor	Deceased donor	SRA overall	Live donor	Deceased donor	MRA overall	
1. Cold ischemia time (minutes)	119 (81- 170) (n=178)	89 (70-118) (n=86)	172.5 (125- 240) (n=60)	113 (80-170)	120 (106.5- 136.5) (n=24)	155 (112.5- 277.5)(n=8)	120 (107.5- 152.5)	<0.001*
2. Warm ischemia time (minutes)	4 (3-7) (n=165)	5 (3-7) (n=85)	4 (3-4) (n=49)	4 (3-6)	5 (4-6) (n=23)	3.5 (3-8) (n=8)	5 (3-6)	0.0127*
Postoperative characteristics								
Serum creatinine (mg/dL)								
At day 0	5.1 (3.72- 6.145) (n=176)	5.1 (3.8-5.9) (n=85)	5.1 (3.95-7.22) (n=60)	5.1 (3.89-6.31)	3.9 (3.4-5.48) (n=23)	5.45 (4.-6) (n=8)	4.34 (3.47-5.5)	0.0691*
At day 5	1.4 (1.08-1.93) (n=179)	1.27 (1.06-1.6) (n=87)	1.64 (1.2-4.5) (n=60)	1.4 (1.1-1.92)	1.28 (1.03-1.66) (n=24)	1.85 (1.08-2.55) (n=8)	1.35 (1.03-1.955)	0.0021*
At day 7	1.4 (1.1-2) (n=178)	1.24 (1.04-1.49) (n=86)	1.68 (1.25-3.36) (n=60)	1.4 (1.1-2)	1.355 (1.1-1.63) (n=24)	1.59 (1.3-2.9) (n=8)	1.45 (1.1-1.84)	<0.001*
At discharge	1.26 (1.05-1.5) (n=181)	1.22 (1.03-1.49) (n=89)	1.4 (1.1-1.64) (n=60)	1.3 (1.04-1.51)	1.12 (1.08-1.45) (n=24)	1.3 (1.14-1.88) (n=8)	1.2 (1.1-1.5)	0.109*
Patient survival (n=182)								
3 months	96.7%	98.86% (n=88)	91.94% (n=62)	96.03%	100% (n=24)	100% (n=8)	100%	0.141^†^
6 months	93.41%	96.59% (n=88)	88.71% (n=62)	93.38%	91.67% (n=24)	100% (n=8)	93.75%	0.237^ †^
1 year	87.91%	92.05% (n=88)	83.87% (n=62)	88.74%	79.17% (n=24)	100% (n=8)	84.38%	0.158^ ‡^
2 year	84.62%	88.64% (n=88)	80.65% (n=62)	85.43%	75% (n=24)	100% (n=8)	81.25%	0.172^ ‡^
Graft survival								
3 months	92.57% (n=175)	96.3% (n=81)	87.1% (n=62)	92.36%	91.67% (n=24)	100% (n=8)	93.75%	0.170^†^
6 months	88.51% (n=174)	91.25% (n=80)	83.87% (n=62)	88.11%	87.50% (n=24)	100% (n=8)	90.63%	0.461^†^
1 year	80.35% (n=173)	84.81% (n=79)	74.19% (n=62)	80.28%	75% (n=24)	100% (n=8)	81.25%	0.181^‡^
2 year	76.88% (n=173)	81.01% (n=79)	69.35% (n=62)	76.06%	75% (n=24)	100% (n=8)	81.25%	0.159^‡^

Subgroup analysis based on donor characteristics (LD vs DD) showed that SRA with DD had a CIT of 172.5min (125-240min), which was longer than that observed in MRA with DD, and this difference was statistically significant (p=0.0001). Table 4 shows other values for each of the groups. The Scr levels of both groups were comparable at various time points in the early postoperative period. On day 5, the median Scr was found to be better controlled in the SRA grafts in both living and deceased grafts (LD=1.27mg/dL (1.06-1.6), DD=1.64mg/dL (1.2-4.5) (p=0.002). On day 7, the median Scr for SRA with DD was higher (1.68mg/dL) in contrast to that in the MRA group with DD (1.59mg/dL); in the case of LD, SRA had a lower Scr (1.24mg/dL) (1.04-1.49) (p=0.0009). SRA with LD (88 patients) was found to have a much higher patient survival rate of 92.05% (81 patients) (p=0.158) and 88.64% (78 patients) (p=0.172) at one- and two-year post-transplant respectively. Graft survival also showed a similar trend. Table 4 shows Scr, graft survival, and patient survival at various time points after transplantation.

Complications that developed postoperatively are summarized in Table 5. The rate of CMV infection was significantly higher in the MRA compared to the SRA (p= 0.000). In addition, within both groups (SRA and MRA), LD grafts were associated with an increased incidence of infections. Among the 63 patients in the SRA with DD group, six (9.52%) patients had DGF and 8.7% (n=2) among the MRA with LD encountered DGF (p=0.173). The MRA with DD patients were found to have a statistically significant higher risk of development of SGF with 12.5% of the eight patients (one patient) (p=0.045) and among the 63 SRA with DD, seven (11.11%) faced SGF with the graft. Table 6 shows that the incidence of post-transplant diabetes mellitus (PTDM) was high in both groups, with seven out of the eight patients undergoing DD with MRAs being diagnosed with PTDM (87.5%) (p=0.13). Four patients had urinary leaks. One patient who underwent LD with four vessels had the ureter completely necrosed and presented with increasing drain output on postoperative day 4. The patient underwent pyelo-urerostomy using a native ureter. Two patients who underwent LD with SRA had urinary leaks, and one patient who underwent DD with SRA also had leaks. All patients were diagnosed with leaks during the immediate postoperative period, and all underwent ureteroureterostomy with a native ureter. Pyelonephritis, acute graft rejections, urinary leak, and renal artery stenosis were some other complications that were seen in patients of SRA and MRA groups, but the difference in these findings was not statistically significant.

**Table 5:** Comparison of postoperative complications in both groups UTI - Urinary Tract Infection *Fisher’s Exact, † Chi-Squared test

Postoperative complications	Overall	Single renal artery			Multiple renal artery			P-value
	(n=185)	Living donor (n=90)	Deceased donor (n=63)	SRA overall	Living donor (n=24)	Deceased donor (n=8)	MRA overall	
Infections								
UTI	24.86%	20%	28.57%	23.53%	25%	50%	31.25%	0.214*
Cytomegalovirus	24.86%	27.78%	12.7%	21.57%	54.17%	0%	40.63%	0.000^†^
BK virus	14.05%	16.67%	12.7%	15.03%	12.5%	0%	9.38%	0.73*
Urological and graft related								
Acute tubular necrosis	20.54%	16.67%	22.22%	18.95%	29.17%	25%	28.13%	0.546^ †^
Acute antibody mediated rejection	14.67%	12.22%	15.87%	13.72%	21.74%	12.5%	19.35%	0.693^†^
Acute cell-mediated rejection	4.89%	2.22%	7.94%	4.57%	8.7%	0%	6.54%	0.219*
Pyelonephritis	13.51%	12.22%	12.7%	12.42%	16.67%	25%	18.75%	0.59*
Mild tubular atrophy	5.95%	7.78%	6.35%	7.19%	0%	0%	0%	0.675*
Delayed graft function	5.43%	2.22%	9.52%	5.2%	8.7%	0%	6.4%	0.173*
Slow graft function	5.43%	2.22%	11.11%	5.8%	0%	12.5%	3.22%	0.045*
Graft hematoma	1.62%	2.22%	1.59%	1.96%	0%	0%	0%	1*
Urinary leak	2.17%	2.22%	1.59%	1.9%	4.35%	0	3.22%	0.671*
Vascular								
Perirenal hematoma	8.11%	3.33%	7.94%	5.23%	20.83%	25%	21.88%	0.01*
Renal artery stenosis	4.32%	2.22%	9.52%	5.23%	0%	0%	0%	0.137*
Pseudoaneurysm of graft renal artery	2.7%	2.22%	4.76%	3.27%	0%	0%	0%	0.558*
Post biopsy hematuria	2.17%	2.22%	3.17%	2.6%	0%	0%	0%	1*
Post Biopsy Hematoma	1.09%	0%	1.59%	0.6%	0%	12.5%	3.2%	0.058*
Anastomotic leak	1.08%	0%	0%	0%	8.33%	0%	6.25%	0.029*
Reanastomosis	0.54%	0%	1.59%	0.65%	0%	0%	0%	0.514*

**Table 6:** Long-term complications developed post-transplant SRA - Single Renal Artery, MRA - Multiple Renal Artery * Chi-Squared test, †Fisher’s Exact

Overall		SRA			MRA			P-value
Long-term complication		Living donor (n=90)	Deceased donor (n=63)	Overall SRA	Living donor (n=24)	Deceased donor (n=8)	Overall MRA	
Post-transplant diabetes mellitus	54.59%	56.67%	46.03%	52.29%	58.33%	87.50%	65.53%	0.13*
New onset hypertension	1.08%	1.11%	1.58%	1.31%	0%	0%	0%	1^†^
Repeat surgery	6.49%	3.33%	12.7%	7.19%	4.17%	0%	3.13%	0.136^†^
Graft dysfunction	27.72%	33.33%	20.63%	28.1%	26.09%	25%	25.81%	0.383*
Nephrectomy	19.02%	17.78%	25.4%	20.9%	8.7%	12.5%%	9.6%	0.332^†^
Incisional hernia	0.54%	0%	1.59%	0.65%	0%	0%	0%	0.514^†^

## Discussion

In this retrospective record-based study of kidney transplant recipients, we found no significant difference in the time Scr took to reach its nadir level after kidney transplantation, regardless of whether the graft had MRA or SRA. There was no difference in long-term graft and patient survival between MRA and SRA grafts. As expected, CIT and WIT were higher with MRA than with SRA. However, there was no significant difference in the incidence of surgical complications between the two groups, except for a higher incidence of perigraft hematoma in the MRA group.

The transplanting of an MRA graft has many inherent problems, such as increased chances of ATN. It can also prolong the ischemia time, thus causing injuries to the graft. Bench reconstruction performed on an MRA graft is the main cause of increased ischemia time. Polar arteries, in particular, are frequently involved in infarctions, infections, and urological complications, thus increasing morbidity and mortality.

The primary objective of this study was to compare the time taken in the post-transplant period for the return of Scr to the nadir level (≤1.0mg/dL). As shown in Table 4, Scr returns to normal values by day 5 post-transplant, and a mild rise is observed in the MRA group on day 7, but this rise was found to be statistically insignificant. In the case of deceased donor transplants in both groups, the Scr levels were on the higher side on postoperative day 5 and day 7, and this difference was found to be statistically significant (Scr at day 5, p=0.0021, Scr at day 7, p=0.009).

When compared in the long term, the graft and patient survival rates were comparable to those in the SRA group. However, the 100% survival rate in the case of MRA with DD may be due to the small size of the group. Hsu et al. also reported findings similar to those of this study concerning one-year postoperative graft survival, immediate post-transplant graft functioning, and complication rates [10]. The MRA group had a longer ischemia time and a higher incidence of post-transplant complications.

The operation time, which includes the WIT and CIT was found to be significantly higher in MRA graft transplant patients [11]. Oh et al. reported a longer WIT and longer hospital stay in the MRA graft group [12]. Post-transplant, the time taken for the graft to start functioning normally, was associated with WIT [11].

Increased ischemia time and shorter time for preparing the recipient for transplantation explain the higher incidence of DGF in DD transplantation. Arpali et al. reported similar rates of DGF in both groups [13]. Mahajan et al. reported a higher incidence of DGF among the MRA group, but it was statistically insignificant [6], our study also establishes similar findings. The increased incidence of DGF in MRA graft recipients may be due to the longer ischemia time, which leads to necrosis of the graft tissue. Another possible reason could be reperfusion injury [14].

In this study, the urological and renovascular complication rates were comparable in both the groups (SRA and MRA); however, the eight patients in the MRA group who underwent DD transplantation faced some urological (or urinary) or renovascular complications. The ureteral complications that develop could be due to the surgical procedure performed to harvest the donor kidney, which involves ureteral devascularization [15]. A record-based study conducted by Ashraf et al. states, “No significant differences were observed among the recipients of all the three groups regarding early vascular and urological complications, post-transplant hypertension, acute tubular necrosis, acute rejection, creatinine level, and graft and patient survival”. In their study, the SRA and MRA graft recipients were classified into three groups based on renal artery multiplicity and the type of anastomosis performed on the recipient vessels [16]. Studies conducted by Aydin et al. did not show a significant difference in patient and graft survival post-operatively [17].

This study has some limitations. First, the study design was a retrospective study based on medical records. This was a single-center study, and the sample size was limited. Transplantation operations were performed by different surgeons and not by the same surgeon. The grafts harvested were from living or DDs, this factor in addition to the open or laparoscopic approach for graft resection could have created the difference in CIT/WIT. Also, the study group (MRA grafts, n=32 patients) compared with the control group (SRA grafts, n=153) was much smaller. On further division into subgroups, the number decreased even further with a group having only eight patients.

## Conclusions

This study highlights that kidney transplants using grafts with MRAs yield outcomes comparable to those with SRA grafts in terms of short-term graft function, long-term patient and graft survival, and post-operative complications. Although MRAs present unique challenges, such as longer ischemia times and a higher incidence of certain complications like perigraft hematomas, the results affirm their feasibility in both living and DD transplants. Notably, these findings suggest that MRAs should not be excluded in donor selection, particularly in regions where expanding the donor pool is essential to meet demand.

Despite its contributions, this study has limitations, including the small size of the MRA cohort and the single-center, retrospective nature of the research. To strengthen these findings, larger, multi-center studies with larger sample sizes are needed to confirm these results and identify ways to reduce complications associated with MRA grafts. Continued advancements in surgical methods and post-operative management will further support the inclusion of MRA donors, which could significantly reduce the burden of ESRD and address the increasing global need for kidney transplants.
